# Role of hypothalamic-pituitary adrenal-axis, toll-like receptors, and macrophage polarization in pre-atherosclerotic changes induced by social isolation stress in mice

**DOI:** 10.1038/s41598-021-98276-2

**Published:** 2021-09-27

**Authors:** Arvin Haj-Mirzaian, Kiana Ramezanzadeh, Siavash Shariatzadeh, Michael Tajik, Farima Khalafi, Armin Tafazolimoghadam, Mahla Radmard, Alireza Rahbar, Fardad Pirri, Kiarash Kazemi, Ayda Khosravi, Niloufar Shababi, Ahmad Reza Dehpour

**Affiliations:** 1 Experimental Medicine Research Center, Tehran University of Medical Sciences, P.O. Box: 13145-784, Tehran, Iran; 2 Department of Pharmacology, Tehran University of Medical Sciences, Tehran, Iran; 3 Department of Pharmacology, School of Medicine, Shahid Beheshti University of Medical Sciences, Tehran, Iran; 4 Department of Pharmacology, Shahid Sadoughi University of Medical Sciences, Yazd, Iran

**Keywords:** Neuroscience, Social behaviour, Social neuroscience, Cardiovascular diseases

## Abstract

It has been well documented that chronic stress can induce atherosclerotic changes, however, the underlying mechanisms is yet to be established. In this regard, this study aimed to elucidate the relation between hypothalamic-pituitary adrenal-axis (HPA-axis), toll-like receptors (TLRs), as well as M1/M2 macrophage ratio and pre-atherosclerotic changes in social isolation stress (SIS) in mice. We used small interfering RNA against the glucocorticoid receptor (GR) to evaluate the relation between HPA-axis and TLRs. C57BL/6J mice were subjected to SIS and RT-PCR, ELISA, flow cytometry, and immunohistochemistry were used to assess the relations between pre-atherosclerotic changes and TLRs, macrophage polarization, pro-inflammatory cytokines, and cell adhesion molecules in aortic tissue. We used TAK-242 (0.3 mg/kg, intraperitoneally), a selective antagonist of TLR4, as a possible prophylactic treatment for atherosclerotic changes induced by SIS. We observed that isolated animals had higher serum concentration of corticosterone and higher body weight in comparison to normal animals. In isolated animals, results of in vitro study showed that knocking-down of the GR in bone marrow–derived monocytes significantly decreased the expression of TLR4. In vivo study suggested higher expression of TLR4 on circulating monocytes and higher M1/M2 ratio in aortic samples. Pathological study showed a mild pre-atherosclerotic change in isolated animals. Finally, we observed that treating animals with TAK-242 could significantly inhibit the pre-atherosclerotic changes. SIS can possibly increase the risk of atherosclerosis through inducing abnormal HPA-axis activity and subsequently lead to TLR4 up-regulation, vascular inflammation, high M1/M2 ratio in intima. Thus, TLR4 inhibitors might be a novel treatment to decrease the risk of atherosclerosis induced by chronic stress.

## Introduction

There is a vast body of evidence indicated that psychological stress, such as social isolation stress (SIS), can be counted as a risk factor of cardiovascular diseases (CVDs) and might increase the risk of morbidity and mortality in CVDs cases^1–4^. Atherosclerosis is the main pathological basis of acute ischemic cardio-cerebrovascular events such as acute coronary syndrome (ACS), myocardial infarction (MI), and ischemic heart disease^5^. Previously, it has been shown that chronic stress can promote the formation of atherosclerosis through excessive activation of the sympathetic nervous system, the renin-angiotensin system, oxidative stress, inflammation, and the hypothalamic–pituitary–adrenal cortex (HPA) axis^6–11^.

It has been well documented that socially isolated animals had higher serum concentration of corticosteroids due to abnormal HPA-axis activity^10,11^. Since glucocorticoid receptors (GR) agonists such as dexamethasone could suppress inflammation through inhibiting the nuclear factor-κB (NF-κB)^12,13^, current study hypothesized that HPA-axis might be the linkage between SIS, innate immune system, and atherosclerosis.

Extensive uptake of lipoprotein and modified lipoprotein, including oxidized low-density lipoprotein (ox-LDL) by differentiated macrophages (derived from circulating monocytes into intima by interactions with their cell adhesion receptors, including P-selectin, E-selectin, intercellular adhesion molecule-1 (ICAM-1), and vascular cell adhesion molecule-1 (VCAM-1)) might result in the formation of lipid-laden macrophage^14,15^.

On the other hand, two major subtypes of macrophages including M1 and M2 macrophages have been strongly linked to the formation of atherosclerosis plaque^16^. It has been demonstrated that M1 macrophages are the most prevalent macrophage in atherosclerosis plaque^16,17^. In addition, it is postulated that polarization of M2 phenotype has an anti-atherosclerotic effect^18^. Interestingly, the polarization of macrophages into M1 or M2 phenotypes has been associated with inflammation and toll-like receptors (TLRs) 2 and 4 signaling pathway^19–21^.

TLRs, as a part of the innate immune system, are the most characteristic members of pattern recognition receptors (PRRs)^22^. Activation of TLRs by various ligands such as pathogen-associated molecular patterns (PAMPs) might result in increased expression of inflammatory cytokines^22^. A few couples of studies have shown that inappropriate activation of TLRs might play a key role in the pathogenesis of atherosclerosis^23^. However, the signaling pathway of the atherosclerosis and excessive activation of TLRs is still unclear.

Previous studies have used genetically modified animals (ApoE knock out mice) to evaluate the role of chronic stress on atherosclerotic plaque formation; however, this non-physiological model could not accurately reflect the human disease. For example, it has been suggested that ApoE knock out mice are expected to have higher levels of very-low-density lipoprotein (VLDL), which is not typical in human atherosclerosis^7^. Therefore, this study could reflect the natural pathophysiology of pre-atherosclerosis changes more accurate than genetically modified animals.

Since the underlying mechanisms of SIS-induced atherosclerosis is yet to be established, this study was aimed to elucidate the effect of SIS on the formation of atherosclerotic plaque in normal mice with high fat diet through M1 and M2 macrophage polarization and evaluate the role of TLR4 signaling pathway and HPA-axis in the recently mentioned subject.

## Material and methods

### Animals and housing conditions

This study used a total of 40 male C57BL/6J mice aged 21–25 days and weighing 20–22 g. Animals were held in two separate conditions: social condition (SC) and isolated condition (IC). The standard laboratory conditions were applied for all animals for a period of 5 weeks (the temperature was 22 ± 2 °C, the humidity was 50 ± 10%, the lights were 12-h dark and free access to food and water). Socially conditioned mice were placed in Plexiglas boxes (25 cm × 25 cm × 15 cm) (6 mice per cage) and isolation conditioned animals were placed individually in Plexiglas boxes (24 cm × 17 cm × 12 cm)^24,25^. In addition, both groups including social and isolation conditioned were fed with high-fat diet (HFD) containing 21% saturated fat and 0.15% cholesterol. The same experimenter washed the cages of isolation conditioned animals regularly to minimize care and social contact. Socially conditioned animals were considered as control group or normal animals in in vivo studies.

### Experimental design and sample acquisition

In this study, we evaluate the impact of SIS on pre-atherosclerotic changes at both level of in-vitro and in vivo. At in-vitro level, we cultured the bone marrow-derived monocyte, which have been obtained from socially isolated animals; then we evaluated the gene expression of TLR2 and TLR4 after knocking-down the GR or NF-κB (p50 subunit) in medium contained dexamethasone.

At in vivo level, isolation conditioned animals were divided into two groups prior to a 5-week period of isolation (IC animals and isolation conditioned animals treated with TAK-242). TAK-242, a selective TLR4 inhibitor, was dissolved in in 1% Dimethyl sulfoxide (DMSO) and then diluted in sterile water^26^. TAK-242 (purchased from Sigma Aldrich Co., LLC) was administrated at a dose of 0.3 mg/kg intraperitoneal twice a week for consecutive 5 weeks of isolation period in isolation conditioned animals based on previous studies^10,24^. TAK-242 and vehicle were administered in the final volume of 10 ml/kg mouse weight.

After a 5-week period of isolation, mice were prepared for the experimental tasks such as electrocardiography assessment. In the next step, after sacrificing animals, the blood samples were obtained and aorta samples, including thoracic aorta, aortic root and aortic sinus were resected and immediately sent for further analysis. In this regard, after sacrificing the mice the thoracic aorta, aortic root, and aortic sinus were harvested. To evaluate pre-atherosclerotic changes in thoracic aorta, aortic sinus, and aortic roots were fixed in 4% paraformaldehyde solution at 4 °C overnight, and then embedded in OCT compound for cryosectioning. Serial cryostat sections 8 μm thick were harvested on slides for further analysis.

### Electrocardiography

For evaluating the cardiac function, we assessed the echocardiography and electrocardiogram (ECG). In this regard, both isolated and normal mice were anesthetized using 2% isoflurane in oxygen and maintained at body temperature. ECG was recorded in normal animals and after the 5-week period of isolation in socially isolated animals. ECG was recorded for 15 min in mice. Needle electrodes attached to a bio-amplifier (ADI Devices, Spain) were implanted subcutaneously for limb lead at position II. The QT interval, QRS complex, and ST segments were assessed for each ECG tracing. The signals were digitized by a Powerlab system at a sampling rate of 10 kHz, and displayed using the software Lab Map 7 (ADInstrument, Australia). The QT intervals, presented as corrected QT (QTc), were calculated in a 5 min ECG. The QTc was obtained using Bazett’s formula $({\text{QTc}}\, = \,{\text{QT}}/\sqrt {\left( {{\text{RR}}/{1}00} \right)} )$.

### Assessment of lipid profile

Total cholesterol, triglycerides (TG), low-density lipoprotein (LDL), high-density lipoprotein levels (HDL) were measured in plasma samples according to manufacturer instructions from Infinity Triglycerides and Wako Chemicals. In brief, plasma samples were mixed with equal volumes of dextran sulfate (20 g/L) and magnesium chloride (1 M) solution pH 7.0 and incubated at room temperature for 10 min followed by centrifugation at 4 °C for 30 min. The supernatant was calculated by means of chromatography to determine the concentration of total cholesterol, free cholesterol, triglycerides, low density lipoprotein (LDL), and high-density lipoprotein cholesterol. All lipid profile components were measured and checked according to instructions from the supplier.

### Assessment of inflammatory cytokines

In this experiment we applied the following measures to assess the molecular assay in target tissue. In this step, after euthanasia, we separated longitudinal axis of thoracic and iliac aorta from the aorta root and the junction of heart. Aorta samples including thoracic aorta, aortic root and aortic sinus were isolated and homogenized in 2 ml of 50 mmol/L phosphate buffer, pH 7.4^10,27,28^. All tissues were divided into equal volume and weight and all homogenized samples had the same volume prior to assessment of inflammatory cytokines. The blood samples were collected in heparinized containers and centrifuged to measure circulating cytokine levels. Pooled plasma from both isolation and social conditioned groups was stored at -80 degrees C until cytokine concentrations were assessed. Samples were centrifuged at 1466×g for 15 min at 4 °C after the samples were homogenized^29^. The TNF-α, IL-6, and IL-1β levels were determined using enzyme-linked immunosorbent assay kit (ELISA; Abcam, Cambridge, MA, USA). The amounts of TNFα, IL-6, and IL-1β were determined according to instructions from the supplier by using a standard curve.

### Real-time PCR analysis

Aorta was resected and immediately frozen by using liquid nitrogen, and stored at − 80 °C. In the first step total RNA was extracted using Trizol reagent (Invitrogen, Cergy Pontoise, France) from the aorta samples. Gene-level alterations in mRNA were determined using qRT-PCR after the reverse transcription of 1 μg of RNA from each sample using PrimeScript RT reagent kit (Takara Bio Inc., Otsu, Japan). By using SYBR Premix Ex Taq Technology (Takara Bio), qRT-PCR was completed on a light cycler device (Roche Diagnostics, Mannheim, Germany). Primer sequences have been designed based on previous reports, and are shown in Table 1. Thermal cycling conditions included an initial activation step for 30 s at 95 °C afterwards 45 cycles as well as a denaturation step for 5 s at 95 °C and a combined annealing/extension step for 20 s at 60 °C^25^. Analysis of the melting curve was conducted to certify if all primers yielded a single PCR product.

**Table 1:** Sequences of primers used for quantitative RT-PCR in mice.

Gene name	Primers (forward and reverse 5′ to 3′)
ICAM-1	F: 5′-TTCACACTGAATGCCAGCTC-3′ R: 5′-GTCTGCTGAGACCCCTCTTG-3′
VCAM-1	F: 5′-CCCAGGTGGAGGTCTACTCA-3′ R: 5′-CAGGATTTTGGGAGCTGGTA-3′
P-selectin	F: 5′-GTCCACGGAGAGTTTGGTGT-3′ R: 5′-AAGTGGTGTTCGGACCAAAG-3′
E-selectin	F: 5′-AGCTACCCATGGAACACGAC-3′ R: 5′-ACGCAAGTTCTCCAGCTGTT-3′
TLR2	F: 5′-CAGCTGGAGAACTCTGACCC-3′ R: 5′-CAAAGAGCCTGAAGTGGGAG-3′
TLR4	F: 5′-CAACATCATCCAGGAAGGC-3′ R: 5′-GAAGGCGATACAATTCCACC-3′
NF-κB	F: 5′-ATGGCAGACGATGATCCCTAC-3′ R: 5′-TGTTGACAGTGGTATTTCTGGTG-3′

### Immunohistochemistry (IHC)

Immunohistochemistry (IHC) has evaluated the expression of macrophage surface receptors in aorta samples, including CD163 (M2 marker) and CD86 (M1 marker). Aorta samples have been resected and washed in 4 steps with 5 min intervals by PBS. In order to retrieve the antigens, sections were then put in 2 normal hydrochloric acid (HCL) solutions for 30 min. In the next step, adding Borate buffer for 5 min neutralized the HCL effect. Samples were then washed with PBS and then a 3% Triton X-100 solution was used for 30 min to permeate the cell membranes, and PBS was again provided for sample cleaning. Goat serum (10.0%) was applied in 30 min to suppress the secondary antibody reaction as an extra color to the background. The samples were then added a primary antibody diluted with PBS (1:100), and the mixture was kept in a refrigerator (2–8 °C) for one day. Then a secondary antibody was added which was diluted with PBS (1:150). The samples were incubated in a 37 °C incubator for one and a half hours. Afterward, 4′,6-diamidino-2-pheny-lindole (DAPI) were added in a dark room and PBS was poured on the samples immediately. Finally, for the cell measurement, the samples were divided into five different areas by using fluorescent Olympus microscope (TET400). Images were analyzed using the ImageJ (Fiji version) software in the next step. Details were obtained of the proportion of positive immunolabeled cells in the total cells in each selected region (the ratio number of positively stained cells/total number of cells × 100).

### Flow cytometry

The blood samples were obtained in heparinized tubes for further study after animal euthanasia in all our experimental tasks. The samples were then centrifuged at 500×g at 4 °C for 10 min, and the supernatants were discarded. The cells were finally resuspended into 3 ml of RBC lysis buffer (for every mouse) and incubated at room temperature for 10 min and washed cells by adding a 47 ml of medium RPMI 1640 and 2% FBS. The cell pellet resuspended with 0.5 ml of RPMI 1640 medium and 2% FBS after centrifuging the samples at 500×g for 10 min at 4 °C and discarding the supernatant^30^. The cells were finally resuspended in 1 mL of PBS, which contained 2% FBS. All samples were eventually stained with antibodies, as mentioned below.

In the next step, all nucleated cells, which were obtained from the previous step were subsequently stained for CD11b (2 ng/ul; eBioscience Ltd., UK) and Ly6C (2 ng/ul; eBioscience Ltd., UK) for 30 min in medium containing 2% FBS for sorting monocytes. Purified and sorted monocytes (CD11b positive with high expression of Ly6C) were evaluated for the expression of TLR2 and 4 by using TLR4 antibody (HTA125, mouse IgG2a) and TLR2 (TL2.1, mouse IgG2a) as well as corresponding isotype antibodies (purchased from eBioscience, San Diego, CA). Cells were then washed after staining with the above-mentioned antibodies, resuspended in RPMI-medium with 2% FBS, and analyzed by using the BD FACSCaliburTM flow cytometer tool and FlowJo version 10.8 software (TreeStar inc.).

### Histopathological study

For histopathological examination, aorta samples were stained with hematoxylin and eosin (H&E). The staining process was carried out according to the protocol below: deparaffinized of the specimens were done at 70 °C for 20 min by using xylene solution, and then rehydrated in two changes of absolute alcohol. Slides is rinsed in hematoxylin solution for 15 min to stain the nuclei, and then washed for 5 min in running tap water. After bluing in 0.2% ammonia water and then washing in tap water for 5 min, the slides were counterstained in eosinphloxine for 1 min and then samples were rinsed in 90%, 96% and 100% ethanol for 2 min. Slides were then embedded in both xylene and mounting media. Eventually, samples were used to monitor inflammation of the tissue and atherosclerotic lesions using an optical microscope (resolution: 40×). All histopathological data were studied by two independent researchers (blinded), and egregious differences were resolved by discussion or omitting data.

In this study, we evaluated the presence of pre-atherosclerotic changes in intima layer such as intimal thickening between IC, SC, and isolation conditioned animals treated with TAK-242. In this regard, we measured the thickness of intima and media layer. For this purpose, we measured the distance between endothelium cells and internal elastic lamina as innermost layer or tunica intima; and we measured the distance between internal elastic lamina and external elastic lamina as tunica media layer.

### Assessment of corticosteroid concentration, body weight and organs weight

To evaluate the basal corticosterone levels, blood sample acquisition was done according to previously published methods^10,11^. Briefly, both socially isolated and normal mice under their basal conditions were decapitated under CO_2_ anesthesia (all procedures were done at 9AM); then immediately blood samples were collected for further analysis^10,11^. Then, all samples were centrifuged at 3000×g for 10 min (4 °C); then the serum samples obtained from previous step were stored at 20 °C until the assay day. Corticosterone levels were measured using a commercial ELISA kit (Biospes, China). In addition, body weight (g) and organs including adrenals (mg) and thymus (mg) were removed and weighted in both socially isolated and normal animals. The corticosteroid level was measured and checked according to instructions from the supplier.

### Isolation and culture of monocytes for in vitro study

Based on our previous study, after sacrificing mice, we resected femur and tibia from surrounding tissue of both normal and socially isolated animals, and then flush the bones with warm medium (5% fetal bovine serum (FBS), + 1% Streptomycin) through a 70 µm nylon^31^. Then centrifuged the samples 250×g for 10 min in 4 °C; and discarded the supernatant and resuspend the cells with approximately 25 ml of medium^32^. We checked and counted the cells in a counting chamber with a light microscope and calculated the number of observed cells. Then we prepared cell for culture, in this regard we seed the cells on 6-well ultra-low-attachment surface plates to prevent permanent adhesion to the bottom of the plate. Use a concentration of 10^6^ cells per ml with up to 6 ml per well and 20 ng/ml M-CSF was used to promote cell differentiation; and culture the cells for at least 5 days at 37 °C in 5% CO_2_. All cells were evaluating to be CD11b positive by using flowcytometry.

### Inhibiting the expression of GR and NF-κB in cultured monocytes using siRNA

In this study, we used siRNA against GR and NF-κB (p50 subunit) to inhibit the expression of GR in cultured monocytes. Both negative control (non-silencing random siRNA) and siRNA against GR and NF-κB (p50 subunit) were purchased from Ambion (Invitrogen, Carlsbad, CA)^33^. During the culturing process in 6-well plates, siRNAs transfected with the concentration of 15 nM using PepMute Plus siRNA transfection reagent (SignaGen Laboratories, Ijamsville, MD), based on the manufacturer’s instruction. Samples were divided into three groups as follow: 1- cells transfected negative control siRNA, 2-cells transfected siRNA against GR, and, 3- cells transfected siRNA against NF-κB (p50 subunit). Cells were allowed to recover for a week in medium with adding 0.01 μmol/L of dexamethasone^33^. After one week of incubation, samples were evaluated for gene expression of NF-κB (p50 subunit), TLR2, and TLR4 by using RT-PCR as mentioned above.

### Statistical analysis

In the current study, One-Way ANOVA and T-test analysis were used to analyze the data by using SPPSS version 25 and graphs were designed by GraphPad Prism version 8. ImageJ was used to interpret and analyze data obtained from IHC. *P* value < 0.05 was as a statistical significance. In this study, we applied Bonferroni *P* value adjustment for RT-PCR result (*P* value less than 0.01 was consider as significance difference). In addition, the sample size was determined using version 3 of G*Power software, considering the study's power of 0.8 and α = 0.05.

### Ethics

Our study was in accordance with the National Institute of Health (NIH) Guidelines for the Care and Use of Laboratory Animals (HHS publication 85-23, 1985), legislation for the protection of animals used for scientific purposes (Directive 2010/63/EU) and also this study was carried out in compliance with the ARRIVE guidelines (PLoS Bio 8(6), e1000412, 2010). All experimental protocols were approved by a Tehran University of Medical Sciences (TUMS) Ethics committee and according to TUMS policies on medical and research ethics (Code: IR.TUMS.VCR.REC.1397.316). Euthanasia was done by using 7 min exposure to CO_2_.

## Results

### Effect of social isolation stress on serum concentration of corticosterone

In this section, we evaluated the serum concentration of corticosterone, body weight, and organs weight including adrenal and thymus in both normal and socially isolated animals (Table 2). T-test analysis showed that socially isolated animals had significantly higher level of corticosterone (42.30 ± 7.67 ng/ml) in comparison to normal animals (33.30 ± 5.03 ng/ml, t = 3.101, df = 18, and *P* < 0.01). Also, we evaluated that the body weight before and after four weeks of experiment in both socially isolated and normal animals. We observed no significant difference between these two groups of mice before starting the experiment animal (IC: 20.32 ± 0.71, SC: 20.58 ± 0.51, t = 0.940, df = 18, and *P* > 0.05). After four weeks, the mean weight observed in socially isolated animals (27.08 ± 1.6 g) was significantly higher than normal animals (25.56 ± 1.1 g, t = 2.476, df = 18, and *P* < 0.05). On the other hand, we did not observed any significant difference in adrenal (IC: 3.51 ± 0.14, SC: 3.49 ± 0.12) and thymus (IC: 57.12 ± 2.8, SC: 58.31 ± 2.6) weights in these two groups (t = 0.343 df = 18, and *P* > 0.05; t = 0.984, df = 18, and *P* > 0.05, respectively).

**Table 2:** Absolute body weight (g) and organ weights (mg) in both isolated and social conditioned mice.

	Groups	
	Social conditioned	Isolation conditioned
Concentration of corticosterone (ng/ml *n* = 10)	33.3 ± 5.0	42.3 ± 7.7**
Body weight before (g, *n* = 10)	20.58 ± 0.51	20.32 ± 0.71
Body weight after (g, *n* = 10)	25.56 ± 1.1	27.08 ± 1.6*
Adrenals (mg, *n* = 10)	3.49 ± 0.12	3.51 ± 0.14
Thymus (mg, *n* = 10)	58.31 ± 2.6	57.12 ± 2.8

*P < 0.05 and **P < 0.01 compared to social conditioned animals.

### Assessment the role of corticosteroids on expression of NF-κB, TLR2, and TLR4

**Figure 1:**
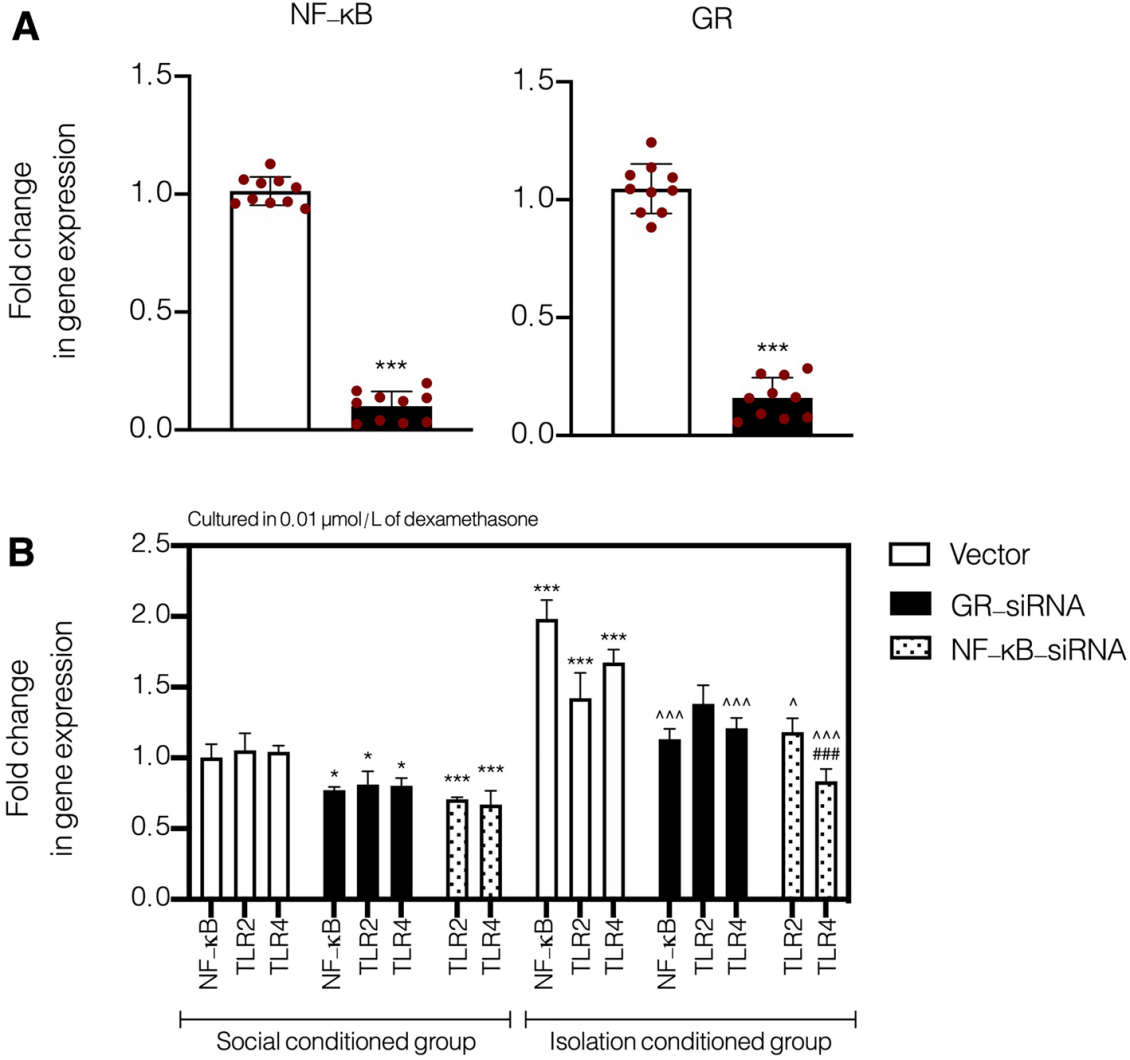
Fold change in gene expression of TLR2, TLR4, and NF-κB in GR- and NF-κB- (p50 subunit) knock-down cultured monocytes derived from bone marrow of socially isolated animals. Values are expressed as the mean ± S.E.M. (n = 10 in each group) results were analyzed using One-Way ANOVA followed by Tukey’s post hoc test. ***P < 0.001 compared to social condition group treated with vector. ^###^*P* < 0.001 compared to isolated condition group treated with GR-siRNA, ^∧^*P* < 0.05 and ^∧∧∧^*P* < 0.001 compared to social isolation group treated with vector.

In order to clarify that hyper activation of GR (in consequence of high serum corticosterone) could impact on the expression of TLR2 and TLR4 on monocytes, we used cultured monocytes derived from both normal and socially isolated animals’ bone marrow. In this section, we hypothesized that hyper activation of GR in socially isolation animals could affect the expression level of TLRs through NF-κB signaling pathway. In this regard, we knock-down the expression of GR and NF-κB (p50 subunit) by using siRNA in cultured monocytes. It should be noted that we checked and verified the inhibitory effects of GR and NF-κB (p50 subunit) siRNAs prior to performing any further investigation (Fig. 1A). T-test analysis showed that both GR-siRNA and NF-κB (p50 subunit)-siRNA could significantly knock-down the expression of GR and NF-κB (t = 24.87, df = 8, and *P* < 0.001, and t = 47.33, df = 8, and *P* < 0.001, respectively).

In this section, we used siRNAs against GR and NF-κB (p50 subunit) in cultured cells extracted from socially isolated animals’ bone marrow. One-Way ANOVA analysis showed that GR and NF-κB (p50 subunit) knocking-down could significantly alter the expression of TLR4 and TLR2 (F (15, 64) = 71.94, *P* < 0.001, Fig. 1B). Post hoc analysis showed that cultured monocytes derived from socially isolated animals had significantly higher expression for TLR4, TLR2, and NF-κB (p50 subunit) in comparison to normal animals (P < 0.001). Also, the expression of both TLR4 and NF-κB (p50 subunit) significantly dropped after knocking-down the GR in the medium contained with 0.01 μmol/L dexamethasone in cultured monocytes derived from socially isolated animals’ bone marrow (*P* < 0.001). The expression of TLR4 decreased significantly after knocking-down the NF-κB (p50 subunit) in comparison to cell treated with GR-siRNA in the medium contained with 0.01 μmol/L dexamethasone (*P* < 0.001). In addition, in comparison to cultured monocytes derived from socially isolated animals’ bone marrow, the expression level of TLR2 remained intact after treating cells with GR-siRNA (*P* > 0.05), however, the expression level of TLR2 significantly decreased after treating cells with NF-κB (p50 subunit)-siRNA in comparison to cultured monocytes derived from socially isolated animals’ bone marrow (*P* < 0.05). On the other hand, our results showed that GR-siRNA could significantly decrease the expression of NF-κB (p50 subunit), TLR2, and TLR4 in cultured monocytes derived from normal animals’ bone marrow in comparison to cells treated with vector (*P* < 0.05); also, results of NF-κB (p50 subunit) knocking down showed significant down-regulation of both TLR4 and TLR4 in cultured monocytes derived from normal animals’ bone marrow in comparison to cells treated with vector (*P* < 0.001). These results showed a tight interaction between GR, NF-κB (p50 subunit), and TLR4 in cultured monocytes derived from both normal and socially isolated animals’ bone marrow. Our in-vitro study showed that abnormal activation of HPA axis and consequently hyper activation of GR in socially isolated animals could result in up-regulation of NF-κB (p50 subunit), TLR4 and TLR2.

### Assessment of plasma level of lipid profile

**Figure 2:**
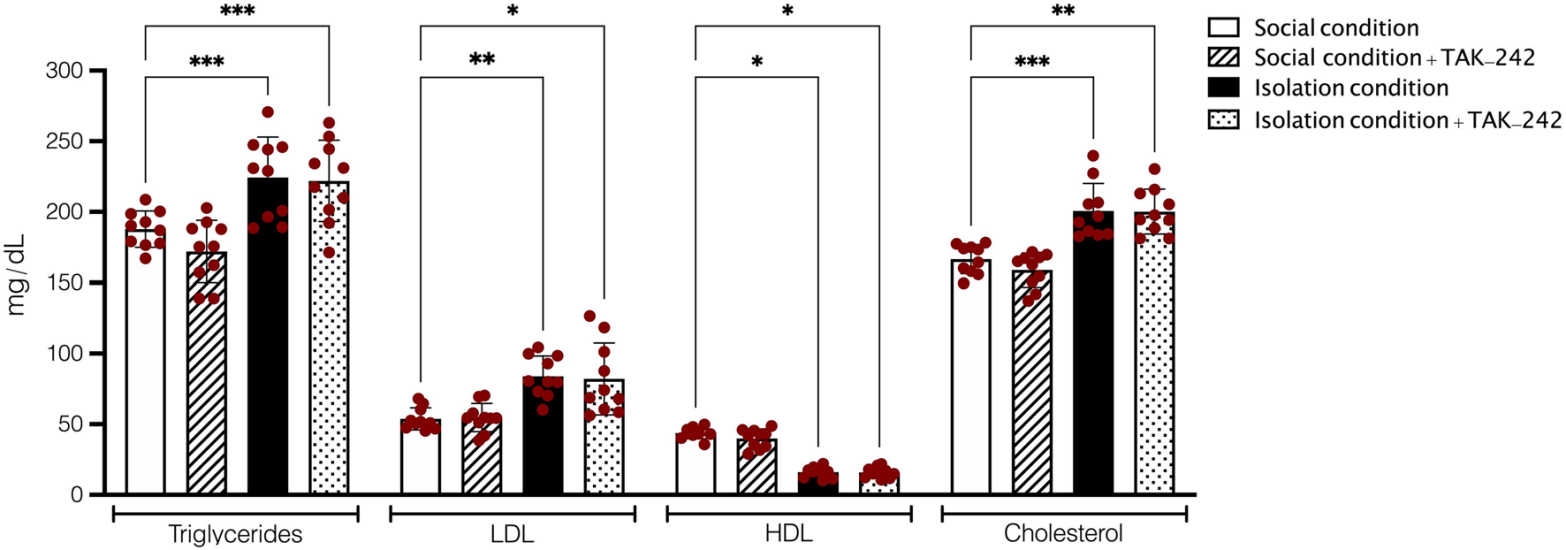
Results of plasma concentrations of lipid profile components in socially isolated and normal animals. Values are expressed as the mean ± S.E.M. (n = 10 in each group) results were analyzed using One-Way ANOVA followed by Tukey’s post hoc test. ***P < 0.001 compared to social conditioned animals.

In the first step of the current study, we assessed the lipid profile in socially isolated animals, as well as normal mice. One-Way ANOVA analysis showed that social isolation significantly impacted on the plasma concentration of HDL, LDL, TG, and cholesterol in comparison to the control group (F (15, 144) = 224.1, *P* < 0.001, Fig. 2). Tukey’s test showed that socially isolated animals had significantly higher plasma levels of LDL, TG, and cholesterol (*P* < 0.01, *P* < 0.001, *P* < 0.01, respectively), in addition to significant lower plasma concentration of HDL in comparison to the control group (*P* < 0.05). However, post hoc test showed that blockage of TLR4 by using administration of TAK‐242 at a dose of 0.3 mg/kg (twice a week) could not significantly alter the impact of SIS on plasma concentration of lipid profile components in socially isolation animal (*P* > 0.05). Also, our results demonstrated that the proportion of LDL to HDL was significantly higher in both isolation conditioned (2.82 ± 0.31) and isolation conditioned animals treated with TAK-242 (3.12 ± 0.56) in comparison to social conditioned mice (1.39 ± 0.22, *P* < 0.001). However, no significant difference was observed between isolation conditioned and isolation conditioned animals treated with TAK-242 (*P* > 0.05); and also, no significant effect was observed in normal animals treated with TAK-242 (*P* > 0.05).

### Evaluation the effect of SIS on expression of TLR2 and TLR4 on the surface of circulation monocytes

**Figure 3:**
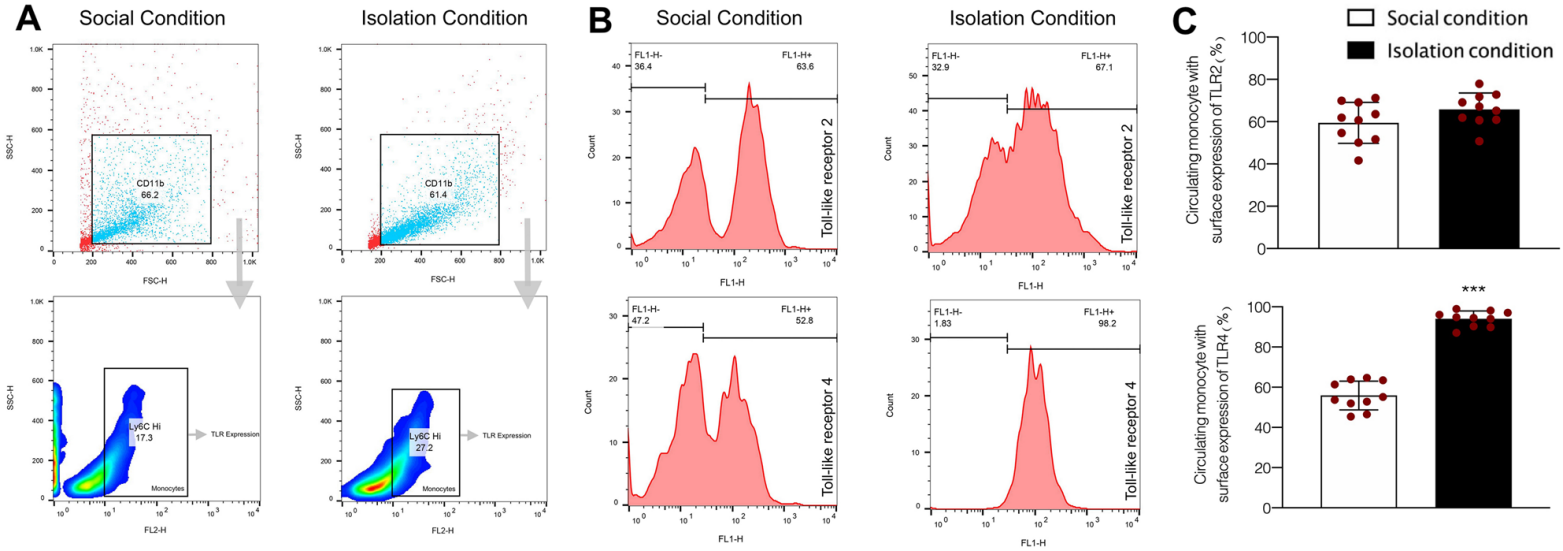
Gating strategy (**A**) and the expression of TLR-2 and 4 on circulating monocytes in both normal and isolated animals (**B** and **C**). Values are expressed as the mean ± S.E.M. (n = 10 in each group) results were analyzed using One-Way ANOVA followed by Tukey’s post hoc test. ***P < 0.001 compared to social conditioned animals.

In this step we tried to evaluate the impact of SIS on the expression of TLR2 and 4 on circulating monocytes by using blood samples. Data obtained from flowcytometry showed that social isolation stress significantly increased the expression of TLR4 on circulating monocytes (t = 0.99, df = 8, *P* < 0.001, Fig. 3). However, t-test analysis failed to show any significant difference between the expression of TLR2 in social condition and isolation condition animals (t = 8.11, df = 8, *P* > 0.05, Fig. 3).

### Evaluation of the leukocyte adhesion receptors gene expression

**Figure 4:**
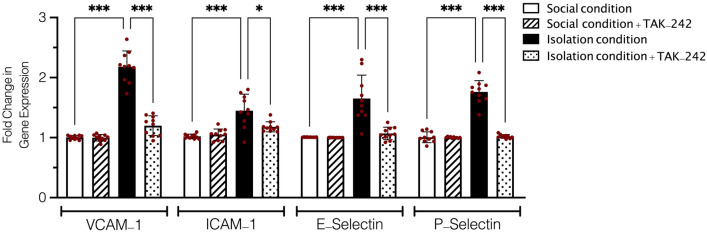
Fold change in gene expression of VCAM-1, ICAM-1, E-selectin, P-selectin receptors among SC, IC, and isolation conditioned animals treated with TAK-242 in aorta samples. Values are expressed as the mean ± S.E.M. (n = 10 in each group) results were analyzed using One-Way ANOVA followed by Tukey’s post hoc test. *P < 0.05 and ***P < 0.001 compared to social conditioned animals. ^#^*P* < 0.05 and ^###^*P* < 0.001 compared to isolation conditioned animals.

Results obtained from One-Way ANOVA analysis showed a significant difference between isolation conditioned animals, socially isolated animals treated with TAK-242, and social conditioned animals in gene expression of VCAM-1, ICAM-1, E-selectin, and P-selectin receptors in aorta samples (F (15, 144) = 49.63, *P* < 0.001, Fig. 4). Tukey’s analysis showed a significantly higher fold change in gene expression VCAM-1, ICAM-1, E-selectin, and P-selectin in isolation conditioned animals in comparison to social conditioned group (*P* < 0.001). On the other hand, socially isolated animals that were treated with administration of TAK-242 at 0.3 mg/kg showed non-significant changes in gene expression of ICAM-1, E-selectin, and P-selectin in comparison to social conditioned animals (*P* > 0.05). In addition, we observed socially isolated animals treated with TAK-242 had significantly lower gene expression of VCAM-1 (*P* < 0.001), ICAM-1 (*P* < 0.05), E-selectin (*P* < 0.001), P-selectin (*P* < 0.001in comparison isolation conditioned animals. Besides, we did not observe any significant changes after administration of TAK-242 in normal animals.

### Evaluation the effect of SIS on macrophage polarization

**Figure 5:**
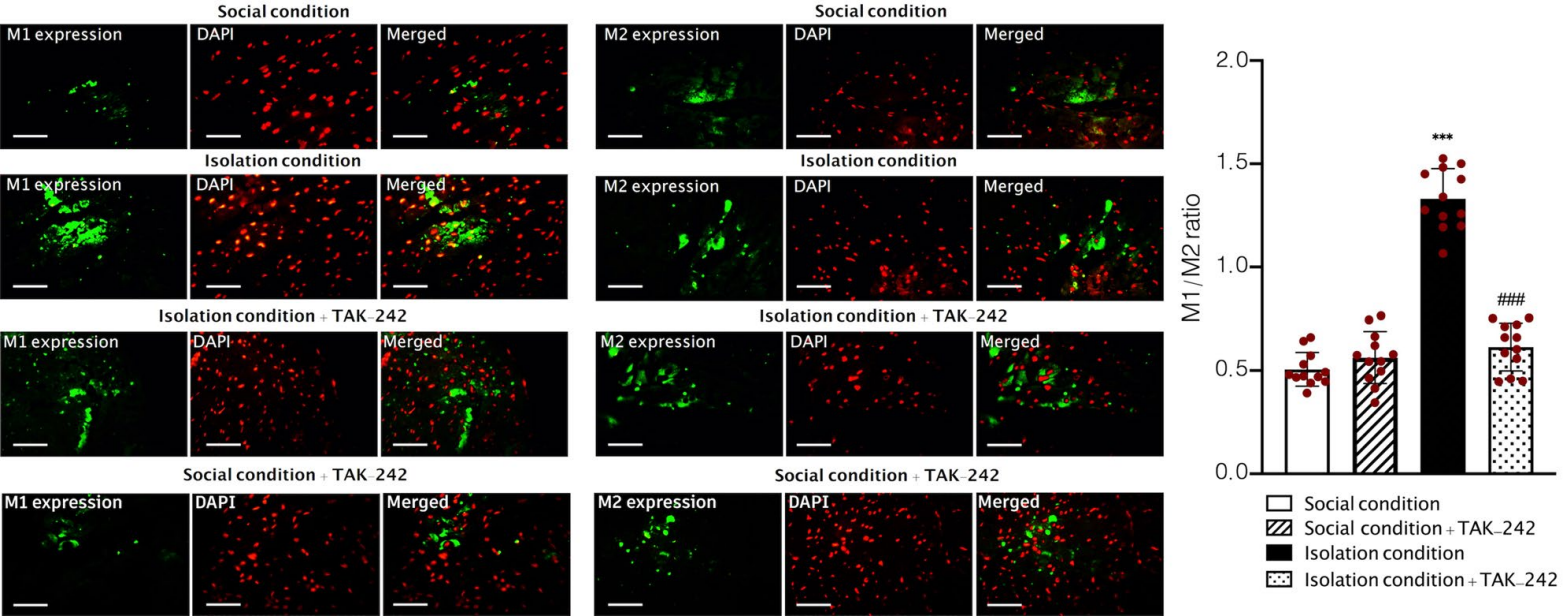
The expression of M1 and M2 biomarkers in aorta samples in social and isolation conditioned animals. Values are expressed as the mean ± S.E.M. (n = 12 in each group) results were analyzed using One-Way ANOVA followed by Tukey’s post hoc test. ***P < 0.001 compared to social conditioned animals. ^###^*P* < 0.001 compared to isolation conditioned animals. Bar = 100 μm.

In the next step, we tried to evaluate the polarization of monocyte-derived macrophages by using immunohistochemistry. One-Way ANOVA analysis showed that SIS significantly impacted on the proportion of M1 to M2 macrophages biomarkers expression (F (3, 44) = 126.0, *P* < 0.001, Fig. 5). Tukey’s test demonstrated that the SIS significantly increased the percentage of macrophages positive for the M2 marker (CD163, *P* < 0.001) in comparison to social conditioned animals. Whereas, administration of TAK-242 twice a week (0.3 mg/kg) in socially isolated animals remarkably decreased the M1/M2 ratio in comparison to isolation conditioned animals (*P* < 0.001), however, no significant effect was observed in normal mice treated with TAK-242 (*P* < 0.05). In addition, post hoc test showed that socially isolated animals treated with TAK-242 had a non-significant M1/M2 ratio difference with normal animals treated with vehicle (*P* > 0.05).

### Assessment inflammatory cytokines

**Figure 6:**
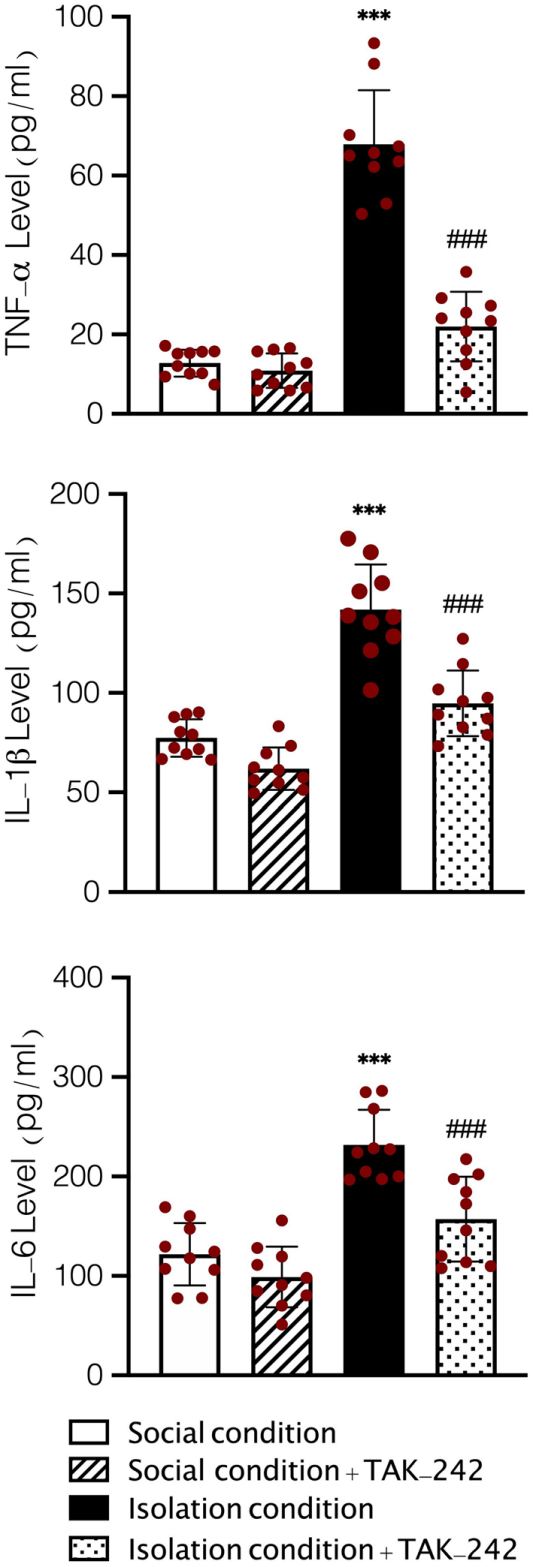
Effects of SIS on concentration of TNF-α (**A**), IL-1β (**B**), and IL-6 (**C**) in aorta samples. Values are expressed as the mean ± S.E.M. (n = 10 in each group) results were analyzed using One-Way ANOVA followed by Tukey’s post hoc test. ***P < 0.001 compared to social conditioned animals and ^###^*P* < 0.001 compared to isolation conditioned animals.

One-Way ANOVA analysis demonstrated that SIS and the blockage of TLR4 could significantly alter the level of TNF-a (F (3, 36) = 98.61, *P* < 0.001, Fig. 6), IL-1β (F (3, 36) = 48.19, *P* < 0.001, Fig. 6), and IL-6 (F (3, 36) = 27.1, *P* < 0.001, Fig. 6) in aorta samples in comparison to social conditioned animals. Tukey’s test showed that SIS significantly increased the concentration of TNF-a in aorta samples in comparison to social conditioned animals (*P* < 0.001). Although, post hoc analysis showed that the mean concentration of TNF-a in aorta samples was significantly higher in socially isolated animals treated with administration of TAK-242 in comparison to social conditioned mice (*P* < 0.001), treating isolation conditioned animals with injection of TAK-242 significantly decreased the concentration of TNF-a in comparison to isolation conditioned mice (*P* < 0.001). We observed the same results in the evaluation of IL-1β and IL-6. Our results showed that SIS significantly increased the level of IL-1β in aorta samples in comparison to social conditioned animals (*P* < 0.001); TAK-242 treatment decreased the level of IL-1β in comparison to isolation conditioned animals (*P* < 0.001). On the other hand, we observed a significantly higher level of IL-6 in aorta samples of isolation conditioned animals in comparison to social conditioned animals (*P* < 0.001), whereas isolation conditioned animals treated with TAK-242 had a significantly lower concentration of IL-6 in aorta samples in comparison to isolation conditioned mice with no treatment (*P* < 0.001).

### Aorta histopathological study

**Figure 7:**
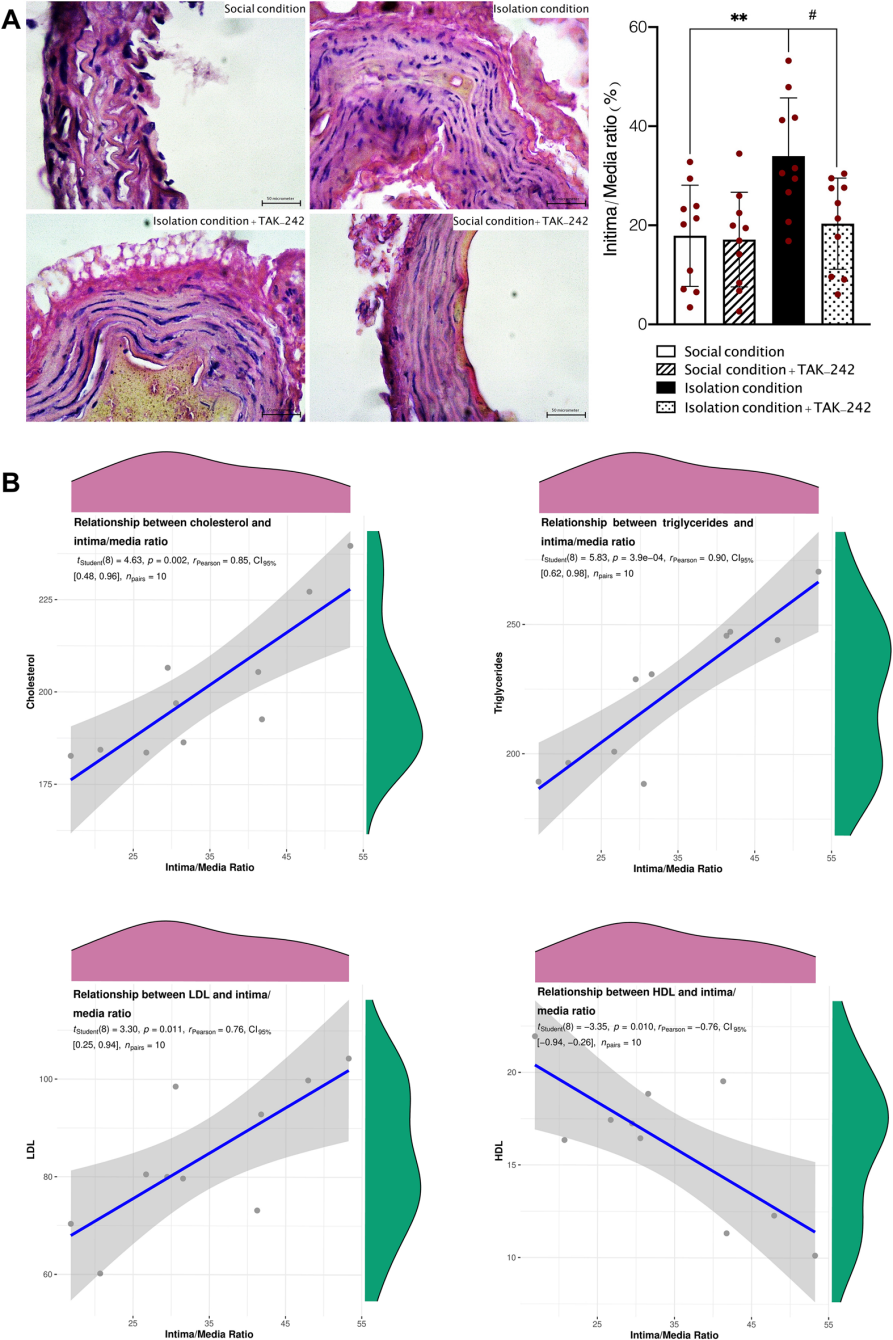
Pre-atherosclerotic changes and intimal/media ratio assessment in social and isolation conditioned animals treated with TAK-242 in comparison to social conditioned animals (40× magnification, **A**); correlation analysis between the level of lipid profile components and intimal/media ratio (**B**). Values are expressed as the mean ± S.E.M. (n = 10 in each group) results were analyzed using One-Way ANOVA followed by Tukey’s post hoc test. **P < 0.01 compared to social conditioned animals. ^#^*P* < 0.01 compared to isolation conditioned animals.

A total of 40 aorta samples from 40 animals, including 10 mice in each group were assessed for pathological study (Fig. 7A). Our results showed a significant difference in the presence of pre-atherosclerotic changes in intima layer such as intimal thickening between IC, SC, and isolation conditioned animals treated with TAK-242. One-Way ANOVA analysis showed that intima/media ratio was significantly higher in socially isolated animals in comparison to normal animals (F (3, 36) = 5.950, *P* < 0.001, Fig. 7). Tukey’s analysis demonstrated that administration TAK-242 could significantly inhibit the intimal thickening and decrease intima/media ratio in socially isolated animals (*P* < 0.05). However, no significant effect was observed after treating normal animals with TAK-242 on intima/media ratio (*P* > 0.05). In addition, we observed that isolation conditioned animals treated with TAK-242 had significantly lower pathological lesions in comparison to isolation conditioned animals. Although TAK-242 was significantly inhibited the intimal thickening, results obtained from histopathological study showed that isolation conditioned animals treated with TAK-242 had a slightly higher presence of pathological lesions in comparison to social conditioned animals. Among all samples in isolation conditioned group, 7 (70%) had pre-atherosclerotic intimal lesions, and 4 (40%) had pathologic intimal thickening. In addition, among samples obtained from isolation conditioned mice treated with TAK-242 at a dose of 0.3 mg/kg, 3 samples (30%) had pre-atherosclerotic intimal lesions, and no sample had significantly pathologic intimal thickening in comparison to animals treated with vehicle. It should be noted that we did not observe any direct and progressive pathological atherosclerotic lesion in all samples.

On the other hand, correlation analysis was performed to evaluate the relation between abnormal lipid profile including TG, LDL, HDL, as well as cholesterol and intima/media ratio in socially isolated animals. Correlation analysis showed that there are significant relations between higher level of LDL, TG, as well as cholesterol and higher intima/media ratio (*P* = 0.011, *P* = 0.003, and *P* = 0.002, respectively, Fig. 7B). Although, we observed statistically significant correlation between higher intima/media ratio and lower HDL (*P* = 0.001, Fig. 7B).

### Evaluation of electrocardiogram

We have assessed the cardiac electrical function among all studied groups in this manuscript. One-Way ANOVA analysis showed that socially isolated animals had a significantly higher heart rate (HR) in comparison to social conditioned animals (F (3, 36) = 6.514, *P* < 0.01, Table 3). However, One-Way ANOVA results failed to show any significant difference among PQ interval duration (F (3, 36) = 0.468, *P* > 0.05, Table 3), corrected QT interval (QTc) duration (F (3, 36) = 0.975, *P* > 0.05, Table 3), and QRS complex duration (F (3, 36) = 0.152, *P* > 0.05, Table 3) in IC, SC, and isolation conditioned animals treated with TAK-242. Post hoc tests showed that HR in isolation conditioned and isolation conditioned animals treated with TAK-242 had significantly higher in comparison to social conditioned mice (*P* < 0.05); however, isolation conditioned and isolation conditioned animals treated with TAK-242 had no significant difference (*P* > 0.05). In addition, we did not observe any arrhythmia among all studied ECGs in all groups.

**Table 3:** ECG results of normal and socially isolated animals.

Items	Social condition (*n* = 10)	Social condition + TAK-242 (*n* = 10)	Isolation condition (*n* = 10)	Isolation condition + TAK-242 (*n* = 10)
HR (bpm)	383 ± 37	377 ± 33	451 ± 47*	443 ± 68*
PQ (msec)	45.9 ± 10.3	48.6 ± 9.9	51.3 ± 12.6	47.8 ± 8.1
QRS (msec)	10.9 ± 0.7	11.1 ± 0.7	11.1 ± 1.8	10.8 ± 1.3
QTc (msec)	42.7 ± 3.5	42.3 ± 2.2	41.8 ± 1.8	40.8 ± 2.7

*HR* heart rate, *bpm* beats per minute, *msec* millisecond. *P < 0.05 compared to social conditioned animals.

## Discussion

In the current study, we examined the possible role of the TLRs, inflammation, and M1/M2 ratio in formation of atherosclerotic plaque in socially isolated animals. Our result showed that SIS could increase the risk of atherosclerosis by enhancing the plasma concentration of total cholesterol, TG, and LDL/HDL. In addition, we observed higher body weight gained in socially isolated animals in comparison to normal animals, but no significant difference was detected between organs weight, including adrenals and thymus, in socially isolated and normal mice. Higher expression of TLR4 on the surface of circulating monocytes in socially isolated animals was observed. Also, higher levels of pro-inflammatory cytokines including TNF-α, IL-1β, and IL-6 in addition to higher M1/M2 were observed in aorta samples of socially isolated animals. The high presence of pre-atherosclerotic lesions in addition to higher intima/media ratio in aorta samples obtained from socially isolated animals have confirmed that SIS could increase the risk of atherosclerosis. In addition, *in-vitro* study demonstrated that HPA-axis abnormal activity in socially isolated animal might increase the expression of TLR4 through NF-κB signaling pathway. Although TLR4 inhibitor (TAK-242) could not induce a significant impact on lipid profile components, TAK-242 significantly inhibited the pre-atherosclerotic changes at both pathological and molecular levels by decreasing the inflammation and M1/M2 ratio. Finally, the electrocardiographic study showed that SIS could significantly increase the heart rate, but no impact on cardiac electrical function.

The impact of social isolation stress on the risk of CVDs might be mediated through stress-related dysregulation of cardiovascular, metabolic, and neuroendocrine processes^34^. In this regard, numerous studies have shown that SIS could significantly induce lipid dysregulation and suggested that social stress could alter lipid metabolism by HPA axis dysfunction and enhancing the transcriptional activity of genes involved in lipid synthesis^34,35^. Gądek-Michalska et al. showed chronic SIS could significantly increase both IL-1β and corticosterone^36^. On the other hand, previous studies demonstrated that corticosteroids suppress the immune response by inhibiting the NF-κB^37^. Although, it has been suggested that the expression of NF-κB can be regulated by TLRs activity, the reverse interaction between them has not been clearly reported. In this study, we observed that SIS increased the serum concentration of corticosterone in vivo; and high level of corticosterone might result in inhibition of NF-κB (p50 subunit) and up-regulation of TLR4 as the first part of this phenomenon Interestingly, upregulation in NF-κB (p50 subunit), TLR2, and TLR4 in daughter cells might suggest a possible epigenetic change in bone marrow-derived monocyte from socially isolated animals. Therefore, further investigation must be conducted to evaluate the role of epigenetic changes in atherosclerosis induced by social isolation stress.

It has been proposed that the levels of total cholesterol, TG, and LDL were markedly higher in animals exposed to chronic unpredictable stress (CUS)^28^. In line with the previous evidence, our results showed that SIS increased the plasma concertation of total cholesterol, and TG, and LDL, as well as increasing LDL/HDL ratio. In addition, previous studies have shown that animals exposed to chronic stressor such as SIS and maternal separation were predisposed to CVDs through mitochondrial dysfunction and increasing cardiac oxidative stress^38,39^. Since ox-LDL plays a key role in the formation of atherosclerosis^40^, a combination of high LDL and oxidative stress levels in vascular tissue could cause an increment in ox-LDL level and subsequently increase the risk of atherosclerosis. This evidence would be the second part of the phenomenon.

It has been well documented that oxidative stress could promote vascular inflammation^41^. In addition, inflammatory response to oxidative stress and increase in the pro-inflammatory cytokines, including TNF-α, IL-1β, and IL-6, has been associated with increasing adhesion molecule expression such as ICAM-1 and VCAM-1^42^. Increase in the expression of these adhesion molecules could increase the rate of monocyte trafficking across the vascular wall^43^. Previous studies showed that social disruption stress led to release of the pro-inflammatory cytokine and increased inflammation in cardiovascular system^27^. In addition, high expression of ICAM-1 and VCAM-1 was observed in CUS^28^. In consistent with previous reports, current study showed that SIS increased the cardiovascular inflammation by enhancing the level of TNF-α, IL-1β, and IL-6. Besides, we showed that the gene expression of adhesion molecules, including ICAM-1, VCAM-1, E-selectin, P-selectin was significantly increased following SIS. Taking together, these results suggested a higher rate of monocyte diapedesis across the vascular wall in socially isolated animals.

TLRs signaling pathway, as an important part of the innate immune system, could orchestrate the adaptive response^44^. Both clinical and experimental reports have demonstrated that the expression of TLR4, TLR1, TLR2, and to a lesser extent TLR5 are involved in formation of atherosclerotic plaques^45^. Results obtained from previous evidence showed that TLR4 and its downstream pro-inflammatory cytokines increased in ApoE^−/−^ mice^10^. In the current study, we observed that circulating monocyte of socially isolated animals have been expressed higher level of TLR4, but not TLR2, in comparison to normal group.

It has been demonstrated that higher expression of TLRs (TLR2, TLR4 and TLR9) can influence lipid uptake by macrophages in intima and increasing the formation of foam cell^46^. On the other hand, it has been reported that TLR signaling pathway may be involved in the regulation of macrophage polarization; excessive activation of TLR4 has been linked to M1 macrophage polarization rather than M2 macrophage phenotype^47^. Interestingly, it has been investigated that a higher M1/M2 ratio is associated with higher risk of atherosclerosis^47^. In this regard, Lee et al. showed that direct injection of inflammatory cytokines accelerated the formation of atherosclerotic plaque through increasing M1 macrophage polarization^47^. In the current study, we observed that macrophages are prone to express M1 phenotype rather than M2 in intima of socially isolated animals in comparison to normal mice.

Previous studies showed that chronic stress can significantly accelerate the atherosclerosis process in ApoE knock out animals. Although, this model helped to investigate the role of high lipid profile in atherosclerotic plaque formation, this non-physiological process can not accurately reflect the human disease. In addition, it has been suggested that ApoE knock out mice are expected to have higher levels of VLDL particles, which is not typical in human atherosclerosis^7^. However, in the current study, normal animals without any genetically modification, which have been encountered with social isolation stress and high-fat diet, have shown pre-atherosclerotic lesions at late adolescence. Thus, this model might be a novel model to evaluate the pre-atherosclerosis process at physiologic and natural condition and is more similar to human plaques formation than genetically modified models.

**Figure 8:**
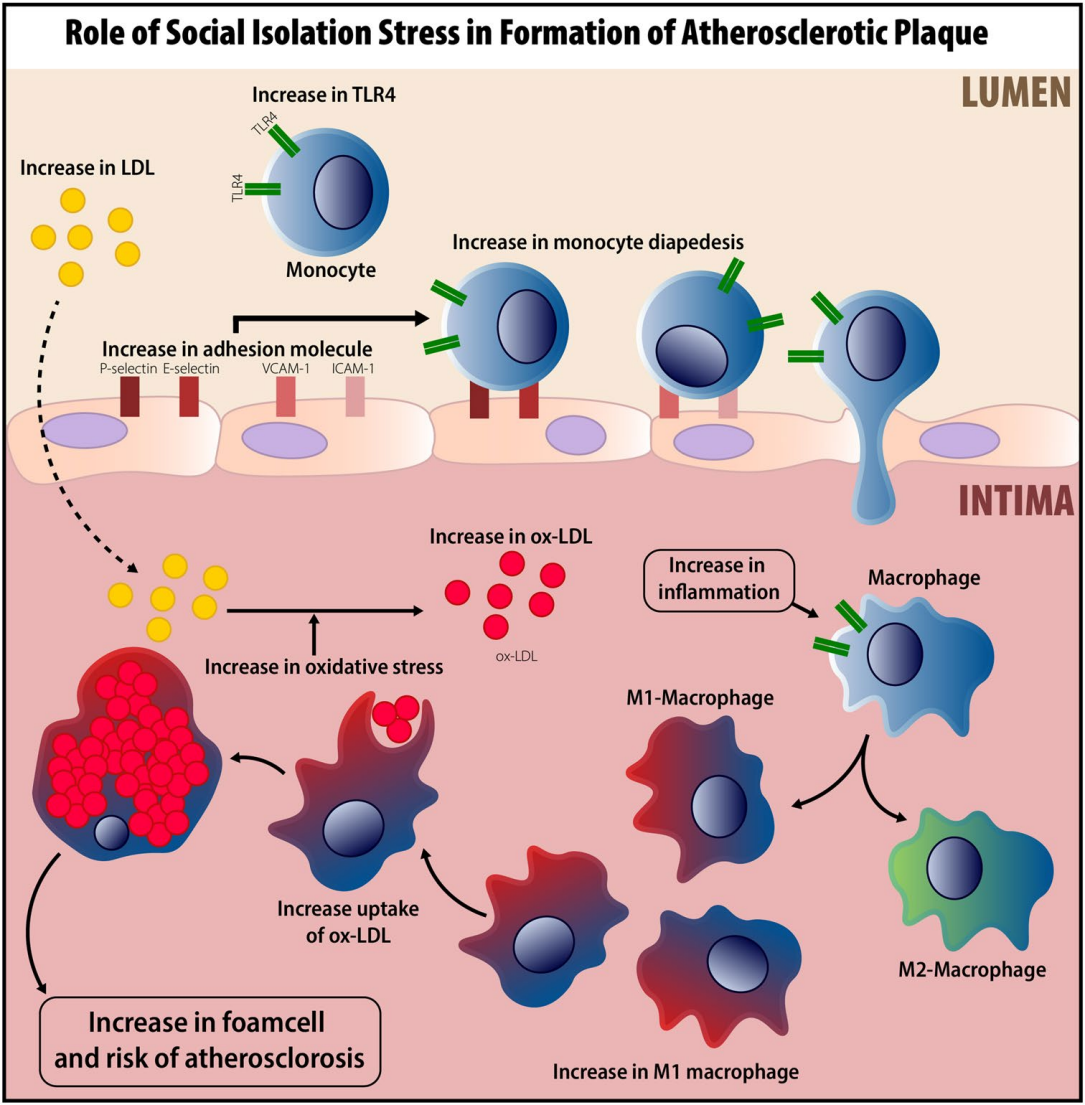
Signaling pathway involved in pre-atherosclerotic changes triggered by social isolation stress.

Conceptual (Fig. 8): (1) SIS induce abnormal activity of the HPA-axis, and high level of corticosterone increase the expression of TLR4 on monocytes through inhibiting of NF-κB signaling pathway (a possible negative feedback from NF-κB to TLR4). (2) SIS increased in lipid profile components, as a risk factor for atherosclerosis process. (3) SIS increased the expression of pro-inflammatory cytokines and adhesion molecules in aorta samples. (4) Based on previous studies SIS could increase cardiac oxidative stress and subsequently increase the level of ox-LDL. (5) SIS increased expression of TLR4 on circulating monocyte. (6) Higher level of TLR4 on circulating monocyte and inflammatory cytokines within aorta samples in addition to higher expression adhesion molecules might increase in diapedesis and shifting to M1 macrophage rather than M2 (increase in M1/M2 ratio), and finally higher uptake of ox-LDL by M1 macrophage might lead to formation of foam cells in intima.

Overall, TLR4 might be a critical part of the pre-atherosclerotic changes to inhibit the formation of atherosclerosis. In this regard, our results showed that inhibiting TLR4 significantly decreased inflammation, M1/M2 ratio, gene expression of cell adhesion molecules, and inhibited the pre-atherosclerotic changes and intimal thickening in histopathological studies. Finally, it should be noted that SIS could not induce a significant impact on the cardiac electrical function, except for the heart rate. It is important to note that this study was used genetically normal animals instead of apolipoprotein E knock-out mice, because evaluating this topic in normal animals is far closer to natural process of atherosclerosis.

This study has several limitations that should be considered. Although western blotting could provide a better insight into the protein expression rather than qRT-PCR, in the current study we did not use western blot due to some limitations. Besides, this study has provided powerful evidence that social isolation stress could induce cardiovascular inflammation, however, there is lack of enough evidence to elucidate that social isolation stress could impact on the level of anti-inflammatory cytokines including IL-10, IL-4, IL-13, and TGF-β. Thus, evaluating the anti-inflammatory cytokines in addition to M2 marker (CD163) in future studies are warranted to provide a better understanding of underlying mechanism of atherosclerosis induce by chronic stress.

## Conclusion

This study showed that SIS could significantly increase the risk of atherosclerosis through HPA-axis, TLR4 up-regulation on circulating monocytes, increased in arterial inflammation, increased in the gene expression of cell adhesion molecules, and finally M1 macrophage polarization in the intima. In addition, we observed that the formation of atherosclerotic plaques might be inhibited by blocking TLR4. In this regard, it could be concluded that TLR4 inhibitors might be a novel treatment to prevent cardio-cerebrovascular events by decreasing the risk of atherosclerosis. Finally, SIS plus high fat diet might be a novel model to evaluate the pre-atherosclerosis process at natural and physiologic condition and is more similar to human plaques formation than genetically modified models.
